# Hepatitis B, hepatitis C and HIV prevalence and related sexual and substance use risk practices among key populations who access HIV prevention, treatment and related services in South Africa: findings from a seven-city cross-sectional survey (2017)

**DOI:** 10.1186/s12879-020-05359-y

**Published:** 2020-09-07

**Authors:** Andrew Scheibe, Katherine Young, Anna Versfeld, C. Wendy Spearman, Mark W. Sonderup, Nishi Prabdial-Sing, Adrian Puren, Harry Hausler

**Affiliations:** 1 TB HIV Care, 11 Adderley Street, Cape Town, South Africa; 2 Department of Family Medicine, University of Pretoria, Pretoria, South Africa; 3 University of Cape Town, Anthropology Section, School of African and Gender studies, Anthropology and Linguistics, Cape Town, South Africa; 4 Division of Hepatology, Department of Medicine, Faculty of Health Sciences, University of Cape Town and Groote Schuur Hospital, Observatory, Cape Town, South Africa; 5 National Institute for Communicable Diseases, 1 Modderfontein Road, Sandringham, Johannesburg, South Africa; 6 Division of Virology, School of Pathology, University of the Witwatersrand, Johannesburg, South Africa

**Keywords:** Hepatitis B, Hepatitis C, HIV, HBV, HCV, People who use drugs, People who inject drugs, Sex workers, MSM, Key populations, South Africa

## Abstract

**Background:**

People who use drugs including people who inject drugs (PWUD/ID), sex workers (SWs) and men who have sex with men (MSM) are at increased risk of HIV and viral hepatitis infection. Limited epidemiological data on the infections exists in key populations (KPs) in South Africa. We investigated the prevalence of hepatitis B (HBV), hepatitis C (HCV) and HIV and selected risk factors among these KPs to inform effective responses.

**Methods:**

We used convenience sampling to recruit a targeted 3500 KPs accessing HIV-related health services across Cape Town (SWs, MSM, PWUD/ID), Durban (SWs, PWUD/ID), Pietermaritzburg (SWs), Mthatha (SWs), Port Elizabeth (SWs), Johannesburg (MSM) and Pretoria (MSM and PWUD/ID) into a cross-sectional survey. An interviewer questionnaire to assess socio-demographic characteristics, drug use and sexual risk practices, was administered. HBV surface antigen (HBsAg); HCV antibody, viral load and genotype, and HIV antibody, was tested.

**Results:**

Among the 3439 people included in the study (1528 SWs, 746 MSM, 1165 PWUD/ID) the median age was 29 years, most participants were black African (60%), and 24% reported homelessness. 82% reported substance use in the last month, including alcohol (46%) and heroin (33%). 75% were sexually active in the previous month, with condom use at last sex at 74%. HIV prevalence was 37% (highest among SWs at 47%), HBsAg prevalence 4% (similar across KPs) and HCV prevalence was 16% (highest among PWUD/ID at 46%).

**Conclusions:**

HBV, HCV and HIV pose a health burden for KPs in South Africa. While HIV is key for all included KPs, HCV is of particular importance to PWUD/ID. For KPs, HBV vaccination and behavioural change interventions that support consistent condom and lubricant access and use are needed. Coverage of opioid substitution therapy and needle and syringe services, and access to HCV treatment for PWUD/ID need to be expanded.

## Background

Viral hepatitis and HIV are significant public health issues. Globally, annual approximated deaths from viral hepatitis are 1.34 million superseding the current 1.3 million from HIV/AIDS [1–3]. This burden is concentrated in sub-Saharan Africa; 25.7 million people are living with HIV [2], 60 million people are chronically infected with hepatitis B (HBV) and 10.2 million people are chronically infected with hepatitis C (HCV) [1]. These three infections share similar transmission routes, particularly exposure to blood and, to a lesser extent, exposure to other bodily fluids [4]. Key populations (KPs) include groups of people at increased risk for acquiring these infections and for their onward transmission, due to a range of structural, social and behavioural risk factors [4]. People who use drugs including people who inject drugs (PWUD/ID), men who have sex with men (MSM) and sex workers (SW) are some of the KPs identified at increased risk for HIV and viral hepatitis [5].

### The South African context

In 2017, 7.9 million people were living with HIV in South Africa [6]. The county’s third National Strategic Plan on HIV, TB and STIs (2017–2022) outlines the steps to be taken to end AIDS [7]. South Africa’s National Hepatitis Guidelines and Action Plan sketch the process for the elimination of viral hepatitis as a public health threat by 2030 [8]. KPs are represented in these policies, which collectively call for tailored prevention, testing and linkage to care services.

A systematic review of 18 studies (136,356 participants) between 1965 and 2013 estimated that South Africa had hepatitis B surface antigen (HBsAg) seroprevalence of 6.7% (95% confidence interval (CI) 6.56–6.83) [9]. A recent household survey in KwaZulu-Natal (a coastal province in the east of South Africa) of 9791 participants, 15–49 years old, revealed an overall HBsAg prevalence of 4.0% (95% CI 3.4–4.5%): 4.8% (95% CI 3.8–5.8%) in men and 3.2% (95% CI 2.5–3.9%) in women (*p* = 0.01) [10]. HBsAg prevalence was 6.4% (95% CI 5.3–7.5%) among HIV-positive participants compared to 2.6% (95% CI 1.9–3.2%) among HIV-negative participants (*p* < 0.01), and was higher among HIV-positive men (8.7, 95% CI 6.3–11.2%) than among HIV-positive women (5.0, 95% CI 3.8–6.2%) (*p* < 0.01) [10]. Despite being vaccine-preventable, many people remain at risk for HBV infection. The hepatitis B vaccine was introduced into the expanded programme of immunisation in April 1995. The country has not implemented a catch-up vaccination programme. Infants born to highly viraemic HBV-infected mothers are particularly at risk. To date, there has not been an HBV mother-to-child transmission prevention programme - neither maternal HBsAg screening nor hepatitis B birth dose vaccination have been provided - but these are now part of National Viral Hepatitis Guidelines [11] with implementation anticipated 1. Most HBV infections in South Africa are horizontally acquired before the age of 5 years [12] (unlike in Asia, where most HBV infections occur perinatally [13]), while HCV and HIV are usually acquired in adulthood [14, 15].

HCV seroprevalence in the general population is estimated to be < 1% [16]. The routine screening of blood and blood products was introduced in South Africa in 1992. Prior to this, most HCV infections were acquired from contaminated blood/blood products with injecting drug use contributing a lesser component [17]. In the last decade, evidence of injecting drug use and the potential spread of HCV through the use of contaminated injecting equipment has been identified in South Africa’s major cities [18]. With the advent of direct acting antivirals (DAAs), HCV can, in most cases, be cured with short courses of all-oral therapy [19]. At the time of writing, no DAAs are registered in South Africa, and are only accessible through a named patient “section 21” certificate process via the South African Health Products Regulatory Authority.

### PWUD/ID

People who use drugs are criminalised, many experiencing stigma and discrimination because of substance use [20]. By the end of 2018, access to needle and syringe programmes was limited to five major metropolitan cities (Cape Town, Durban, Johannesburg, Port Elizabeth and Pretoria) [21] and opioid substitution therapy (OST) services to Cape Town, Durban, Johannesburg and Pretoria [22]. These services were implemented by civil society organisations. Services did not provide other injecting equipment (for example, cookers) necessary for limiting the spread of blood borne infections, or routine HBsAg or HCV screening and further management [20]. Reliable estimates of the number of people in South Africa who use non-regulated or illicit drugs do not exist. Based on regional data (2018), the United Nations Office on Drugs and Crime estimates the annual prevalence of substance use among adults (15–64 years) to be 1% for amphetamine-type stimulants (which includes methamphetamine), 1% for cocaine and 0,5% for opioids [23]. Epidemiological data on blood borne infections among PWUD/ID in South Africa has been limited, mostly focusing on people who inject drugs (PWID). Local modelling data and expert consensus suggests there to be between 67,000 and 75,000 PWID in the country [24, 25], with evidence pointing towards increasing injecting practices [26]. HIV prevalence among PWID was 14% in 2013 [18]. A small study (*n* = 271 PWID) in 2012 found an HCV seroprevalence of 24% and HBsAg seroprevalence of 7% [27]. As we have published elsewhere [28] we found an HIV prevalence of 21%, HBsAg seroprevalence of 5% and a HCV seroprevalence of 55% among 943 PWID from 3 cities.

### MSM

Despite a constitutionally progressive approach to same sex practices, the HIV burden among the estimated 1.2 million MSM is high [29]. HIV prevalence across the main metropolitan areas ranges from 18% in Bloemfontein to 48% in Durban [30, 31]. Less data is available on viral hepatitis among MSM. A small study among MSM in Cape Town identified an HCV seroprevalence of 27% (*n* = 41) and HBsAg prevalence of 2%. Among those MSM with positive HCV antibody tests, 80% reported having injected heroin or methamphetamine in the previous 3 months [32]. Another Cape Town metropolitan based study among HIV-infected MSM identified HCV seroprevalence of 6% (*n* = 285) [33]. HIV prevention and treatment programmes for MSM are well established in most urban settings [34], however, little focus has been placed on the integration of viral hepatitis services.

### SWs

There are an estimated 150,029 (137,641 female, 6882 male and 5506 transgender) SWs in South Africa [35]. Sex work remains illegal and related discrimination in communities and healthcare facilities limits access to health services and undermines effective disease prevention and care [36]. HIV prevalence among female SWs is particularly high ranging from 37% in Cape Town to 75% in Durban [37], and 88% in Pietermaritzburg [38]. Very little data on HIV prevalence among male or transgender SWs exist and data on viral hepatitis among SWs is unavailable.

People may relate to more than one KP grouping. For example, an earlier study among PWID ﻿(*n* = 450) found that 25% of male participants had ever had sex with another man and 9% had ever worked as a sex worker [18]. Between 11 and 53% of MSM in major urban areas have sold sex to other men [31]. In contrast, a bio-behavioural survey among female SWs in three cities (2013–2014) highlighted that less than 0.5% of female SWs had ever injected a drug [39].

This study was designed to investigate the prevalence of HBV, HCV and HIV and their co-infections and to describe selected risk factors among KPs in seven South African cities.

## Methods

The selection of cities, populations of focus and sample size were based on implementing partners’ service delivery platform,^2^ available resources and relative size of each city. Prospectively, we aimed to recruit 3500 participants from 11 sites across the seven cities. This included 1550 SWs (400 each in Cape Town and Durban, cities with populations above 1,5 million based on the latest census (2011) [40] and 250 each in Pietermaritzburg, Port Elizabeth and Mthatha, smaller cities); 750 MSM (250 each from Cape Town, Pretoria and Johannesburg); and 1200 PWUD/ID (400 each in Cape Town, Durban and Pretoria). There were more SW sites due to the larger SW HIV and health service programme relative to other KP programmes at the time. PWUD/ID were recruited from cities that operated harm reduction services at a PWUD:PWID ratio of 1:4 due to the increased risk of blood borne infection transmission through injecting practices. Fewer (250) MSM were recruited in each city, due to the existence of some data for this population and due to the limited resources available.

Consenting participants self-defined as being 18 years or older and having met the following criteria, as relevant, in the past 12 months i) SW: having exchanged sex for money^3^; ii) MSM: having been born biologically male and having had penetrative or receptive oral or anal sex with another male; iii) PWUD: having used heroin, cocaine or methamphetamine and iv) PWID: having injected a substance for non-therapeutic purposes, irrespective of the type of drug injected or the mode of injection.

Convenience sampling was used with the study conducted as an extension of existing HIV prevention, sexual health and harm reduction services provided by non-profit organisations targeting KPs in the respective cities. Each of the service providers operate dedicated teams that provide targeted services to specific KPs they already served. In Cape Town, for example, routine health services for SWs and PWUD/ID were provided by a single service provider out of a single physical location, but were provided in parallel by different mobile teams and were designated as two distinct study sites recruiting SW or PWUD/ID respectively. Although there is alignment of services, each site tended to have separate clients depending on how the clients themselves best identified. Participants recruited through a SW site and who met inclusion criteria would have been counted as a SW irrespective of substance use behaviour and vice versa for PWUD/ID. Similarly, at MSM sites, people accessing HIV testing and/ or treatment services were informed about the study and invited to participate and would have been recruited as MSM. Refreshments were offered during the last month of the study’s recruitment period to encourage enrolment at PWUD/ID sites to meet the recruitment targets within the time frame allocated for fieldwork.

﻿Data collection took place between August 2016 and October 2017. In all cases, a study team member administered a standard, confidential health-screening questionnaire to participants who provided written informed consent. The questionnaire captured data on demographics (age, sex, race and housing), substance use (substances used in the last month and injecting in the last month, including injecting frequency, needle and syringe reuse and sharing), use of opioid substitution therapy and sexual risk behaviour (condom use, penile-vaginal intercourse, anal intercourse, transactional sex and substance use in the context of sex) and prior health screens and previous HIV testing.

The paper-based questionnaire was completed in English and administered in private spaces. Where closed rooms were not possible, questionnaires were completed out of earshot of other people and then filed and locked away. The questionnaire captured participants’ names, surnames, dates of birth and included a participant identification code. Participants were not obligated to provide identifying information. Service providers and study staff were all trained in ethical research practices and signed research confidentiality agreements. Access to participant names and surnames was restricted to clinicians providing health services and staff conducting the questionnaire. All other study documentation, including blood samples, used unique participant identification codes.

For HIV, HBV and HCV point-of-care (POC) testing, blood was drawn. POC HIV testing was done in line with national protocols [41]. POC HBsAg testing was performed using the Determine™ HBsAg (Alere Inc., MA, USA). POC HCV testing was done using OraQuick® HCV rapid antibody test (Orasure Technologies Inc., PA, USA). Samples that were HCV POC reactive were sent to the National Institute for Communicable Diseases (NICD) in Johannesburg for anti-HCV testing (ARCHITECT Anti-HCV assay) on the ARCHITECT i1000SR system (Abbott Laboratories, Diagnostics Division, IL, USA). The COBAS® AmpliPrep/COBAS® TaqMan® HCV Quantitative Test v2.0 on the COBAS® Ampliprep Taqman® Analyzer (Roche Molecular Systems, CA, USA) was used for quantitative assessment of HCV antibody positive samples. PCR amplification and HCV genotyping was performed using the Versant LiPA amplification and Versant HCV genotyping 2.0 assays (LiPA, Innogenetics, Ghent, Belgium), respectively.

Study team members provided participants with appropriate counselling, condoms and lubricant, and PWID received sterile injecting equipment. Participants who screened positive for HBV or who were diagnosed with confirmed HIV or HCV infection were referred to a pre-identified health facility for work-up and/or assessment for treatment. HBV vaccination was offered to all clients who were HBsAg POC negative^4^.

Anonymised data was imported into STATA 14.0 (Statacorp LLC, Texas, USA) for data analysis. Data was analysed using descriptive statistics (proportions, medians and inter quartile range (IQR)), stratified by KP. Age was also categorised based on age groups used by the Joint United Nations Programme on HIV/AIDS, with a binary cut off value of 25 years [42]. Racial groupings were based on those used by Statistics South Africa [43]. POC seroprevalence was calculated for HIV and HBsAg. HBsAg seroprevalence was also calculated for people born before and after the introduction of childhood HBV vaccination in 1995. Anti-HCV prevalence was based on laboratory results. The HCV viraemic rate is the number of participants with detectable HCV RNA with laboratory based anti-HCV antibody confirmation.

The study was approved by the Human Research Ethics Committee of the University of Cape Town (ref: 004/2016), the Research Ethics Committee of the University of the Witwatersrand (ref: M160510) as well as the Eastern Cape (ref: EC_2016RP19_818), Western Cape (ref: WC_2016RP19_818) and KwaZulu-Natal (ref: KZN_2016RP59_986) Provincial Department of Health Ethics Committees. Participants did not receive any remuneration.

## Findings

In total, 3509 participants were recruited and enrolled. Seventy participants were excluded from the per protocol analysis. Reasons for exclusion included: ineligibility (did not meet criteria for KP) (*n* = 23), study informed consent form could not be located (*n* = 11), source documents missing (*n* = 4), error in testing procedure for HBV and/or HCV or test not done (*n* = 32). In a per protocol analysis, 3439 KP were included (1528 SWs, 746 MSM, 1165 PWUD/ID).

### Socio-demographics

Table 1 outlines socio-demographics. Participants’ median age was 29 (IQR 25–35) years. Most participants were black (60%, 2078/3439). SWs were almost exclusively female, in all locations other than Cape Town, where male SWs were recruited (12%, 47/284). PWUD/ID were predominantly male (85%, 999/1175). Almost a quarter of participants (23%, 807/3439) were between the ages of 18 and 24 years. Age did not vary particularly between KP sub-groups – with 2 exceptions: in Mthatha, 42% (104/248) of SWs were under 25 years of age and in Cape Town PWUD/ID were older, with only 8% (28/367) under 25 years. PWUD/ID disproportionately reported homelessness; ranging from 55% (201/367) in Cape Town to 70% (278/398) in Durban. In Cape Town, SWs also reported higher levels of homelessness (17% (66/384)) than were reported in other SW groups.

**Table 1:** Participants’ socio-demographic characteristics (*n* = 3439)

	SWs (***n*** = 1528)					MSM (***n*** = 746)			PWUD/ID (***n*** = 1165)			TOTAL
	CT	PE	Mthatha	DBN	PMB	CT	JHB	PTA	CT	DBN	PTA	
**N**	384	248	248	399	249	250	250	246	367	398	400	3439
**Age**												
Median (IQR)	31 (26–37)	28 (24–35)	26 (23–30)	30 (25–35)	27 (24–31)	31 (26–39)	29 (24–34)	29 (24–37)	31 (28–35)	27 (25–31)	30 (26–34)	29 (25–35)
< 25 years	80 (21%)	78 (31%)	104 (42%)	90 (23%)	78 (31%)	49 (20%)	68 (27%)	65 (26%)	28 (8%)	96 (24%)	71 (18%)	807 (23%)
≥ 25 year	304 (79%)	170 (69%)	144 (58%)	309 (77%)	171 (69%)	201 (80%)	182 (73%)	181 (74%)	339 (92%)	302 (76%)	329 (82%)	2632 (77%)
**Sex**												
*Female*	337 (88%)	247 (> 99%)	248 (100%)	397 (> 99%)	246 (99%)	0	0	0	69 (19%)	42 (11%)	55 (14%)	1641 (48%)
*Male*	47 (12%)	1 (< 1%)	0	2 (< 1%)	3 (1%)	250 (100%)	250 (100%)	246 (100%)	298 (81%)	356 (89%)	345 (86%)	1798 (52%)
**Race**^**a**^												
*Black*	106 (28%)	182 (73%)	247 (99%)	369 (92%)	249 (100%)	102 (41%)	197 (79%)	118 (48%)	15 (4%)	218 (55%)	275 (69%)	2078 (60%)
*Coloured*	247 (64%)	49 (20%)	0	12 (3%)	0	47 (19%)	9 (4%)	9 (4%)	274 (75%)	45 (11%)	13 (3%)	705 (21%)
*White*	18 (5%)	17 (7%)	0	5 (1%)	0	92 (37%)	37 (15%)	109 (44%)	71 (19%)	96 (24%)	109 (27%)	554 (16%)
*Indian/ Asian*	1 (< 1%)	9	0	10 (3%)	0	4 (2%)	2 (1%)	4 (2%)	1 (< 1%)	39 (10%)	1 (< 1%)	62 (2%)
*Other*	12 (3%)	0	1 (< 1%)	3 (1%)	0	5 (2%)	5 (2%)	6 (2%)	6 (2%)	0	2 (1%)	40 (1%)
**Homeless**	66 (17%)	1 (< 1%)	0	0	0	14 (6%)	1 (< 1%)	3 (1%)	201 (55%)	278 (70%)	262 (66%)	826 (24%)
**General population**^**b**^	3,740,026	1,152,115	451,710	3,442,361	618,536	3,740,026	4,434,827	2,921,488	3,740,026	3,442,361	2,921,488	

^a^Based on race categories used by Statistics South Africa. Coloured is a South African term which refers to people of mixed ancestry. The national distribution of race is: Black 80,9%, Coloured 8,8%, White 7,8%, Indian/ Asian 2,5% [43]

^b^Population of municipality based on most recent census (2011)

*CT* Cape Town, *DBN* Durban, *JHB* Johannesburg, *PE* Port Elizabeth, *PMB* Pietermaritzburg, *PTA* Pretoria

### Substance use

Overall, 82% (2818/3439) of participants reported use of at least one substance in the previous month. Alcohol was the most commonly reported substance used (46%, 1568 / 3439), and use was higher among SWs and MSM than among PWUD/ID. Heroin (known locally as nyaope or whoonga) was the most frequently reported illegal/unregulated substance used in the last month (33%, 1138/3439) followed by methamphetamine (14%, 486/3439), cannabis (13%, 433/3439), cocaine (6%, 204/3439) and methcathinone (2%, 68/3439) (Table 2). Almost all PWUD/ID had used heroin in the last month, ranging from 82% (300/367) in Cape Town to 99% (394/398) in Durban. Methamphetamine use in the last month was highest in Cape Town across all three sub-groups: 80% of PWUD/ID (293/367), 28% of SWs (107/384) and 11% of MSM (27/250). PWUD/ID living in Pretoria had the highest reported use of cannabis (30%, 121/400) and cocaine (30%, 118/400). This was substantially higher than that reported for any other KP sub-group or region for all three substances. Cannabis use in other KPs was variable, ranging from zero among SWs in Mthatha to about one-fifth of MSM in Johannesburg.

**Table 2:** Substance use practices among participants (*n* = 3439)

	SWs (***n*** = 1528)					MSM (***n*** = 746)			PWUD/ID (***n*** = 1165)			TOTAL
	CT	PE	Mthatha	DBN	PMB	CT	JHB	PTA	CT	DBN	PTA	
**N**	384	248	248	399	249	250	250	246	367	398	400	3439
**Substances used in last month**												
*Any substance*^a^	296 (77%)	216 (87%)	194 (78%)	305 (76%)	160 (64%)	176 (70%)	199 (80%)	119 (48%)	361 (98%)	396 (99%)	396 (99%)	2818 (82%)
*Alcohol*	217 (56%)	205 (83%)	194 (78%)	295 (74%)	148 (59%)	152 (61%)	192 (77%)	102 (42%)	29 (8%)	19 (5%)	15 (4%)	1568 (46%)
*Heroin*^b^	32 (8%)	1 (< 1%)	0	0	12 (5%)	17 (7%)	3 (1%)	1 (< 1%)	300 (82%)	394 (99%)	378 (95%)	1138 (33%)
*Methamphetamine*	107 (28%)	17 (7%)	0	2 (1%)	1 (< 1%)	27 (11%)	11 (4%)	6 (2%)	293 (80%)	8 (2%)	14 (4%)	486 (14%)
*Cannabis*	55 (14%)	14 (6%)	0	32 (8%)	9 (4%)	39 (16%)	43 (17%)	31 (13%)	45 (12%)	44 (11%)	121 (30%)	433 (13%)
*Cocaine*	12 (3%)	0	0	31 (8%)	4 (2%)	11 (4%)	4 (2%)	2 (1%)	9 (2%)	13 (3%)	118 (30%)	204 (6%)
*Methcathinone*	4 (1%)	2 (1%)	0	20 (5%)	0	8 (3%)	11 (4%)	10 (4%)	1 (< 1%)	1 (< 1%)	11 (3%)	68 (2%)
**Injected a drug in the last 12 months**	0	0	0	0	0	24 (10%)	9 (4%)	8 (3%)	292 (80%)	315 (79%)	313 (79%)	961 (28%)
**Injected a drug in last month**	0	0	0	0	0	22 (9%)	8 (3%)	8 (3%)	290 (79%)	313 (79%)	311 (78%)	952 (28%)
**Currently on OST for ≥ 30 days**	0	0	0	0	0	7 (3%)	1 (< 1%)	1 (< 1%)	12 (3%)	16 (4%)	21 (5%)	58 (2%)

^a^Refers to any of the substances listed in this table

^b^Heroin is known locally as nyaope and whoonga

*CT* Cape Town, *DBN* Durban, *JHB* Johannesburg, *PE* Port Elizabeth, *PMB* Pietermaritzburg, *PTA* Pretoria

In the complete cohort, 28% reported injecting a drug in the last month (952/3439). Among PWUD/ID, close to 80% reported injecting a drug in the last month. No SWs reported having injected any drug in the last month; in this group the reported heroin use was through smoking or inhalation. Injecting in the last month among MSM ranged from 3% in Johannesburg and Pretoria (8/250 and 8/236, respectively) to 9% in Cape Town (22/250). In Cape Town, proportionately more MSM last injected methamphetamine than PWUD/ID (18% (4/22) versus 6% (15/243)) and injected at a lower frequency (64% (14/22) injecting less than 4 times a day, versus 74% (179/243) injecting 4 or more times per day, respectively). A similar pattern was seen among MSM reporting injecting in the last month in Pretoria compared to male PWUD/ID counterparts, with 75% (6/8) MSM reporting to have last injected methamphetamine while 99% PWUD/ID (268/270) last injected heroin. Most Pretoria MSM who injected, reported less frequent injecting when compared to male PWUD/ID with 63% (5/8) injecting less than 4 times a day, compared to 73% of PWUD/ID (196/ 270) injecting 4 or more times per day.

Five percent (54/1138) of all participants who reported having used heroin in the last month also reported taking some form of OST for the last 30 days or more.

### Sexual risk practices

Sexual activity in the past month was reported in 75% (2589/3439) of all participants - highest among SWs (98%) (Table 3). MSM reporting sexual activity in the last month varied from 69% (169/246) in Pretoria to 79% (197/250) in Cape Town. Sexual activity was lowest among PWUD/ID; ranging from 29% (114/400) in Pretoria to 55% (217/398) in Durban.

**Table 3:** Sexual risk practices (*n* = 3439)

	SWs (***n*** = 1528)					MSM (***n*** = 746)			PWUD/ID (***n*** = 1165)			Total
	CT	PE	Mthatha	DBN	PMB	CT	JHB	PTA	CT	DBN	PTA	
**N**	384	248	248	399	249	250	250	246	367	398	400	3439
**Sexually active in the last month**	376 (98%)	244 (98%)	245 (99%)	396 (99%)	248 (> 99%)	197 (79%)	189 (76%)	169 (69%)	194 (53%)	217 (55%)	114 (29%)	2589 (75%)
**Number of people had sex with in last week, if sexually active (median, IQR)**	5 (3–10)	3 (2–6)	2 (1–5)	8 (5–10)	4 (2–6)	1 (1–1)	1 (0–1)	1 (0–1)	1 (1–1)	1 (1–1)	1 (1–1)	2 (1–5)
**Penile-vaginal sex in last week**	361 (96%)	240 (97%)	246 (> 99%)	397 (100%)	245 (98%)	8 (3%)	9 (4%)	10 (4%)	178 (49%)	208 (52%)	100 (25%)	2002 (59%)
**Condom used at last penile-vaginal sex among those who had penile-vaginal sex in last week**	339 (93%)	147 (61%)	81 (34%)	397 (100%)	230 (94%)	6 (75%)	4 (44%)	3 (30%)	95 (53%)	122 (59%)	61 (61%)	1485 (74%)
**Receptive anal intercourse in last week**	49 (13%)	7 (3%)	9 (4%)	82 (21%)	9 (4%)	91 (36%)	76 (30%)	81 (33%)	4 (1%)	0	3 (1%)	411 (12%)
**Condom used at last receptive anal sex among those who had receptive anal intercourse in last week**	41 (84%)	7 (100%)	0	81 (99%)	7 (78%)	53 (58%)	45 (59%)	51 (63%)	2 (50%)	0	3 (100%)	290 (71%)
**Lubricant used at last receptive anal sex among those who had receptive anal intercourse in last week**	30 (61%)	1 (14%)	0	76 (95%)	5 (56%)	71 (78%)	67 (88%)	72 (90%)	2 (50%)	0	1 (33%)	325 (80%)
**Drugs/ goods received in exchange for sex in last month**	40 (10%)	137 (55%)	204 (82%)	3 (1%)	26 (10%)	10 (4%)	7 (3%)	1 (< 1%)	17 (5%)	2 (1%)	10 (3%)	457 (13%)
**Money received in exchange for sex in last month**	363 (95%)	212 (85%)	244 (98%)	388 (97%)	240 (96%)	13 (5%)	9 (4%)	3 (1%)	18 (5%)	7 (2%)	16 (4%)	1513 (44%)
**Alcohol or substance use at last sex**	253 (66%)	124 (50%)	133 (54%)	290 (73%)	11 (4%)	65 (26%)	87 (35%)	54 (22%)	129 (35%)	5 (1%)	162 (41%)	1313 (38%)

*CT* Cape Town, *DBN* Durban, *JHB* Johannesburg, *PE* Port Elizabeth, *PMB* Pietermaritzburg, *PTA* Pretoria

Among SWs, almost all (96–100%) had engaged in penile-vaginal sex in the last week. Condom use at last penile-vaginal sex was lowest in Mthatha (34%, 81/248) and over 90% in Cape Town, Durban and Pietermaritzburg (93%, 339/384; 100%, 397/397 and 94%, 230/249, respectively). PWUD/ID reports of penile-vaginal sex in the last week was lowest in Pretoria (25%, 100/400) and highest in Durban (52%, 208/398). Among these, reported condom use at last penile-vaginal sex was 61% (61/100) and 59% (122/208) in Pretoria and Durban, respectively.

Receptive anal intercourse was reported by 12% (411/3439) of participants overall. Very few (< 1%, 7/1165) PWUD/ID reported receptive anal intercourse. One third (33%, 248/746) of MSM reported receptive anal intercourse in the last week. Of these, 60% (149/248) reported condom use at last receptive anal sex with 85% (210/248) reporting the use of lubricant. A total of 156 SWs reported receptive anal sex in the last week, highest in Durban (21%, 82/399). Seventeen male SWs reported anal sex in the last week, all from Cape Town. Condom use at last receptive anal sex in SWs was generally high, between 78% (7/9) in Pietermaritzburg to 99% (81/82) and 100% (7/7) in Durban and Port Elizabeth, respectively. None of the nine women engaging in receptive anal sex in Mthatha reported condom use. Lubricant use at last receptive anal sex act varied from none in Mthatha to 95% (76/82) in Durban.

Overall, 38% (1313/3439) of participants reported substance use at last sex. This was generally highest among SWs (53%, 811 / 1528), who mainly reported using alcohol, with those in Durban reporting the highest prevalence (73%, 290/399). Among PWUD/ID, substance use at last sex ranged from 1% (5/400) in Pretoria to 35% (129/398) in Durban. Among MSM, substance use at last sex was more consistent varying between 22% (54/246) in Pretoria to 35% (87/250) in Johannesburg.

### Access to HIV services

Overall, for 11% (373/3439) of study participants it was the first time that they had ever had a health screen and been tested for HIV. PWUD/ID had the highest levels of first health screens and HIV tests, with the majority of their previous contacts being linked to accessing sterile injecting equipment. A quarter of all participants (25%, 867/3439) reported to be HIV-infected at the time of participation, ranging from 2% among PWUD/ID in Cape Town and Pretoria (9/367 and 9/400, respectively) to 62% (154/249) among SWs in Pietermaritzburg. The proportion of people living with HIV reporting current antiretroviral therapy (ART) was highest among MSM recruited from MSM health clinics (93%, 255/274) and lowest among PWUD/ID recruited largely as part of community outreach activities (41%, 21/51) (See Table 4).

**Table 4:** Health care access (*n* = 3439)

	SWs (***n*** = 1528)					MSM ***n*** = 746)			PWUD/ID (***n*** = 1165)			Total
	CT	PE	Mthatha	DBN	PMB	CT	JHB	PTA	CT	DBN	PTA	
**N**	384	248	248	399	249	250	250	246	367	398	400	3439
**First health-screen that includes HIV testing**	19 (5%)	2 (1%)	2 (1%)	0	1 (< 1%)	15 (6%)	2 (1%)	3 (1%)	58 (16%)	86 (22%)	184 (46%)	372 (11%)
**Self-reported HIV + ve**	18 (5%)	67 (27%)	109 (44%)	194 (49%)	154 (62%)	90 (36%)	110 (44%)	74 (30%)	9 (2%)	33 (8%)	9 (2%)	867 (25%)
**Self-reported in care on ART, if self-reported HIV-infected**	8 (44%)	54 (81%)	93 (85%)	168 (87%)	134 (87%)	87 (97%)	97 (88%)	71 (96%)	1 (11%)	12 (34%)	8 (89%)	733 (84%)

*CT* Cape Town, *DBN* Durban, *JHB* Johannesburg, *PE* Port Elizabeth, *PMB* Pietermaritzburg, *PTA* Pretoria

### Testing results

Details of the testing results are provided in Table 5. HIV prevalence was 47% (711/1528) among SWs, 43% (320/746) among MSM and 19% (227/1165) among PWUD/ID. HIV prevalence was highest among SWs in Pietermaritzburg at 80% (199/249) and lowest among PWUD/ID in Cape Town (6%, 22/367). There was considerable variation in prevalence of HIV between cities among SWs and PWUD/ID but it remained fairly similar among MSM.

**Table 5:** HIV, HBsAg, anti-HCV, HCV PCR prevalence and HCV viraemic rate among participants (*n* = 3439)

	SWs (***n*** = 1528)					MSM (***n*** = 746)			PWUD/ID (***n*** = 1165)			TOTAL
	CT	PE	Mthatha	DBN	PMB	CT	JHB	PTA	CT	DBN	PTA	
**N**	384	248	248	399	249	250	250	246	367	398	400	3439
**HIV + ve**	69 (18%)	90 (38%)	133 (56%)	220 (55%)	199 (80%)	101 (40%)	122 (49%)	97 (41%)	22 (6%)	65 (16%)	140 (35%)	1258 (37%)
**HBsAg + ve**	13 (3%)	8 (3%)	10 (4%)	17 (4%)	13 (5%)	9 (4%)	10 (4%)	6 (2%)	20 (5%)	15 (4%)	20 (5%)	141 (4%)
**Anti-HCV + ve ELISA**	1 (< 1%)	0	0	0	0	16 (6%)	3 (1%)	1 (< 1%)	136 (37%)	113 (28%)	288 (72%)	558 (16%)
**HCV viral load detectable**	0	0	0	0	0	15 (6%)	2 (1%)	0	102 (28%)	90 (23%)	227 (57%)	436 (13%)
**HCV viraemic rate (among anti_HCV + ve)**	0	0	0	0	0	94%	67%	0	75%	80%	79%	78%
**Anti-HCV-HIV co-infection**	0	0	0	0	0	6 (2%)	3 (1%)	1 (< 1%)	10 (3%)	25 (6%)	113 (28%)	158 (5%)
**Anti-HCV prevalence among HIV + ve participants**	0	0	0	0	0	6 (6%)	3 (2%)	1 (1%)	10 (45%)	25 (38%)	113 (81%)	158 (13%)
**Anti-HCV-HBV co-infection**	0	0	0	0	0	1 (< 1%)	0	0	5 (1%)	5 (1%)	17 (4%)	28 (< 1%)
**HIV-HBsAg co-infection**	5 (1%)	3 (1%)	9 (4%)	13 (3%)	12 (5%)	5 (2%)	7 (3%)	3 (1%)	3 (1%)	6 (2%)	9 (2%)	75 (2%)
**HIV-anti-HCV-HBV co-infection**	0	0	0	0	0	0	0	0	0	2 (1%)	8 (2%)	10 (< 1%)

*CT* Cape Town, *DBN* Durban, *JHB* Johannesburg, *PE* Port Elizabeth, *PMB* Pietermaritzburg, *PTA* Pretoria

HBsAg positivity was 4% (141/3439), ranging from 2% (6/246) among MSM in Pretoria to 5% among PWUD/ID in Cape Town (20/367) and Pretoria (20/400) and SWs in Pietermaritzburg (13/249). HBsAg positivity was 1% (3/307) among people born after 1995 (the year that childhood immunisation was implemented) and 4% (138/3132) among those born before 1995.

In PWUD/ID, anti-HCV ranged from 28% (113/318) in Durban to 72% (288/400) in Pretoria, with an HCV viraemic rate of 80% (90/113) and 79% (227/288), respectively. Six percent (16/250) of MSM in Cape Town had detectable anti-HCV antibodies, among whom all but one had detectable HCV RNA. Only one SW was anti-HCV antibody positive but not viraemic. The overall viraemic rate was 78% (436/558).

Overall, 5% (158/3439) were HCV-HIV co-infected, and was highest among PWUD/ID in Pretoria (28%, 113/400).

Thirteen percent (158/1258) of HIV-infected study participants were HCV co-infected; highest among PWUD/ID in Pretoria (81%, 113/140).

The overall prevalence of HCV-HBV co-infection was less than 1, and 2% (75/3439) were HIV-HBV co-infected. Ten participants were HCV-HBV-HIV triple-infected, all of whom where PWUD/ID.

Among male SWs reporting anal intercourse in the last week (all of whom were from Cape Town), 41% (7/17) were HIV infected, 6% (1/17) HBV infected, one person had HIV-HBV co-infection and no HCV infections were detected. Among 4 male PWUD/ID reporting anal intercourse in the last week, half were either HCV or HIV infected with one HIV-HCV co-infected; none were HBV infected.

Of the 38 participants recruited from MSM sites reporting injecting drugs in the last month, HCV prevalence was 37% (14/38) [45% (10/22) in Cape Town, 38% (3/8) in Johannesburg and 13% (1/8) in Pretoria], HIV prevalence was 37% (14/38) [23% (5/22) in Cape Town, 63% (5/8) in Johannesburg and 50% (4/8) in Pretoria)] and HBV prevalence 3% (1 infection in Cape Town).

Median HCV viral load was 5.45 log_10_ IU/ml (IQR 4.81–5.95). PWUD/ID had a higher median viral load compared to MSM, which was not statistically significant (5.38 versus 5.18 log_10_IU/ml, *p* = 0.3). Genotyping was achieved in 92% (400/435). The most prevalent HCV genotypes were 1a (74%, 296/400) and 3a (14%, 56/400). Mixed genotype infections were seen in 11 PWUD/ID with most from Cape Town (6/11). Genotype 4 was found only in MSM. Of note, no genotype 5 was detected (see Table 6).

**Table 6:** HCV genotype among participants with detectable HCV viral load (*n* = 400)

	SW	MSM	PWUD/ID	Total
**Genotype 1**				**314**
1	0	1	11	12
1a	0	12	284	296
1b	0	0	6	6
**Genotype 3**				**71**
3	0	1	11	12
3a	0	0	56	56
3c	0	0	3	3
**Genotype 4**				**4**
4	0	2		2
4a/4c/4d	0	2		2
**Mixed genotype**				**11**
1a and 3	0	0	1	1
1a and 3a	0	0	8	8
1a and 3c	0	0	1	1
1b and 3a	0	0	1	1
**Total**	**0**	**18**	**382**	**400**

## Discussion

This is the largest and most comprehensive investigation of HBV, HCV and HIV among KPs in South Africa to date. The study confirms earlier research highlighting the high HIV burden among these populations [16]. Most importantly, it documents the alarmingly high seroprevalence of HCV infection among PWUD/ID (46%).

### Demographics

This study follows South African census distinctions of race. These remain relevant because education, socio-economic status and socio-cultural norms continue to map onto apartheid-era categories to some extent. In 2015, 81% of the population self-defined as Black, 9% as Coloured, 8% as White and 3% as Indian/Asian. The Western Cape has the highest proportion of people classified as Coloured (50% of the population) [43], partially explaining the proportionally higher number of Coloured people in Cape Town in each demographic category included in this study.

MSM were recruited from existing, specialised fixed site sexual health clinics. These served people from a wide range of socio-economic circumstances, reflected in the comparatively higher number of white MSM (up to 44% in Pretoria) included in the study and the lower rates (1 to 6%) of people who reported homelessness.

SWs were recruited through a combination of fixed sites and mobile services. Nationally, 5% of SWs are estimated to be male, a figure relatively stable across provinces [35] but this was not reflected in the study (< 1% SWs male). Low numbers of SWs reporting homelessness may be because income generated from SW provides a means of paying for accommodation. Another explanation may be that current SW services are not reaching homeless SWs.

PWUD/ID were largely recruited at locations where mobile needle and syringe distribution and collection services where provided. The higher numbers of men who use drugs included in this study reflects programmatic data that indicates higher numbers of men (between 87 and 90%) to be using harm reduction services [44]. The low proportion of females who use drugs in this and other local studies [17, 44] is likely also reflective of the gender distribution of PWUD/ID. Female-specific barriers to services – including a lack of tailored services and higher rates of stigma and discrimination experienced by women who use drugs [45] – likely also affected the number of female PWUD/ID in this study. The need for gender-appropriate and specific HIV and HCV services for women who use drugs is increasingly recognised locally [46], regionally [47] and globally [48]. The high levels of homelessness among PWUD/ID likely reflects the socio-economic circumstances of PWUD/ID who used the services and/or who were accessible by the study team. PWUD/ID with financial means could access prevention commodities and supplies from pharmacies and/or through the private healthcare system.

### HBV

The overall HBsAg prevalence of 4% is similar to the general population [9, 45] with a marked difference between those born before and after the introduction of HBV childhood immunisation (4% versus 1%). This finding provides additional evidence supporting the benefit of the introduction of childhood HBV vaccination in 1995. However, some people born after this date have acquired HBV infection. The overall coverage of the 3-dose HBV vaccine schedule in South Africa was estimated to be 74% in 2016 [49], and the birth dose vaccine to prevent mother-to-child transmission is yet to be implemented.

### HCV prevalence

The HCV findings are anticipated yet alarming, despite the study bias towards KPs who access HIV prevention, treatment and related services. Globally, HCV is a particular concern among PWID [50], with high prevalence being documented in Africa, ranging from 30% among PWID accessing opioid substitution therapy in Tanzania [51] to 97% of PWID in Mauritius [52]. Our findings show PWUD/ID carried the highest HCV burden with some marked geographic variations. The extremely high rates of HCV seroprevalence in PWUD/ID in Pretoria (72%) is possibly due to the relatively longstanding nature of this injecting community, with sub-optimal access to prevention interventions [53]. Viraemic rates at the upper limit (75–80%) of what would be anticipated may point towards repeated infections [46]. Equally, HCV seroprevalence in MSM, with high HIV-HCV co-infection prevalence, is expected given the risks of HCV infection among MSM, especially in HIV positive MSM and MSM with a history of injecting drug use [32]. The almost non-existent HCV seroprevalence among SW is supported by the low rate of injecting drug use and high condom use in this sample.

The circulating prevalence of HCV genotypes 1a and 3a is similar to other countries where HCV infection is networked and predominantly spread through the sharing of contaminated injecting equipment among PWID [54, 55]. However genotype 5a (a unique and prevalent genotype) was not found. A study of blood samples from patients attending specialist clinics (*n* = 941) and blood donors (*n* = 294) between 2008 and 2012 identified genotype 5a to be common among specialist clinic patients (36%) and particularly common (60%) among people over 50 years old in both groups, and among Black African people (54%) [56]. The lack of genotype 5a in this study suggests that the modes of transmission of identified genotypes among PWUD/ID and other KPs differs from older patients attending specialist clinics, some of whom contracted HCV through blood transfusions and unsafe medical injections [57], traditional practices such as tribal scarification [58] or unknown risks.

### HIV

Lower levels of self-reported HIV status compared with measured prevalence (25 and 37% respectively) has been documented previously in South African KPs [59]. The fact that 25% of participants indicated that this was their first health-screening (including HIV testing) may indicate that a large number of people were genuinely unaware of their status due to fears of testing or competing priorities. This discrepancy may also relate to reporting bias and internalised stigma, fear of knowing one’s status and fears of acknowledging positive status while not on ART [60].

In Cape Town and Pretoria, HIV prevalence was notably higher among the few MSM reporting recent injecting compared to their male PWUD/ID counterparts. The different HIV prevalence may be linked to the recruitment of MSM from well-established clinics that have been providing HIV treatment for MSM for several years, and the elevated risk among MSM who engage in multiple high-risk practices.

Overall reported use of ART, at 84%, was below the 90% target for people living with HIV, but higher than the national average of 57% [61], likely a consequence of the fact that participants were accessed through HIV service delivery platforms. The wide range across cities and populations (from 11% in PWUD/ID in Cape Town to 96% MSM in Pretoria) also likely reflects which services are being primarily accessed at the included organisations by the target populations. The relatively lower levels of ART coverage reported among PWUD/ID (51%) further likely reflects the numerous barriers affecting ART uptake [20] in this population.

### Blood borne virus co-infections

A global systematic review and meta-analysis of the prevalence and burden of HCV co-infection in people living with HIV reported a 6% coinfection prevalence in MSM and 82% in PWID compared to 2% within the general population [62]. In comparison, our findings indicate 3% HCV co-infection prevalence in MSM living with HIV and 65% in PWUD/ID living with HIV. A South African study among antenatal attendees living with HIV found HCV prevalence of 0.1% [63].

Overall 2% were HIV-HBV co-infected with similar prevalence in all 3 KPs.

The number of participants who were co-infected with HIV/HBV or HIV/HCV, or HCV-HBV-HIV triple-infected is cause for concern given the extent to which co-infections can accelerate and amplify disease [5]. There is therefore the need for screening for co-infections in KPs especially HIV-infected PWUD/ID.

Viral hepatitis awareness programmes that emphasise the risk of co-infections and reinfection and provide prevention strategies need to be up-scaled across all cities in South Africa for all KPs. The importance of preventative HBV vaccination needs to be stressed and routine access to vaccination at all levels of care is essential.

### Risk practices

Risks for these blood-borne infections are exacerbated by a host of social and structural factors including limited access to opioid substitution therapy and needle and syringe services for PWID; criminalisation of sex work and drug use and corresponding marginalisation and difficulty accessing healthcare (for PWUD/ID and SWs) and homelessness (largely PWUD/ID).

#### Substance use

In addition to alcohol, the high reported use of heroin is of concern. Though the majority of heroin used in South Africa is smoked [44], it is also the most commonly injected drug among PWID [28]. The HIV and viral hepatitis risks related to heroin are largely through injecting, with heroin dependant people injecting on average four times per day [28]. Methamphetamine use is more common in Cape Town than in the other cities. HIV and viral hepatitis risks relating to methamphetamine use are either indirect, through sexual risk as a result of increased sexual risk taking, or direct, through injecting [64], which has been documented in Cape Town and is not uncommon [28].

A small proportion of MSM across the three cities reported injecting drug use, mostly methamphetamine. The prevalence and patterns of heroin and/or methamphetamine injecting among MSM has not been well documented in South Africa [32]. The injection of methamphetamine in the context of sexual encounters (*Chemsex*) has been documented among sub-groups of MSM [65]. Chemsex among MSM can be associated with unprotected anal intercourse, and in some instances with multiple sex partners. This can be continued for long durations (up to days), and has been identified as a risk factor for HIV and viral hepatitis in other countries [66, 67].

No SWs recruited reported injecting, however, networks of SWs who inject drugs have been reached through more recent harm reduction and sex worker programmes operating in Johannesburg.^5^ Risks involved in substance use are exacerbated by the criminalisation of drug use, limited access to harm reduction services, and discriminatory treatment within healthcare service settings [68].

#### Sexual practices

Sexual practices, unsurprisingly, differed considerably between KP sub-groups. SWs were more likely to report recent sexual activity, multiple partners, transactional sex and substance use at last sex as well as having the highest reported condom use, in general. Local research among female SWs in South Africa has identified high reported levels of condom use in major metropolitan areas (89% in Cape Town and 84% in Durban) [39].

SWs in this study were least likely to report lubricant use in relation to receptive anal sex. Little data exists on the use of lubricant during anal sex among SWs. Data from a three city bio-behavioural survey among female SWs in Cape Town, Johannesburg and Durban showed that only a quarter of female SWs were aware of lubricant, with low levels of reported use [39].

The greatest regional variation in sexual practices was also seen among SWs. Those in the Mthatha region were more likely to engage in high-risk sex, with lower condom use and a greater propensity to engage in sex in exchange for goods and commodities. In contrast, those working in KwaZulu-Natal, reported much higher condom use and almost exclusively received money in exchange for sex. This may suggest lower negotiating power of sex workers in Mthatha and could explain the markedly lower rates of condom use at last penile-vaginal sex reported there. Elsewhere, penile-vaginal condom usage in the general population has been shown to correlate to education and exposure to behaviour change interventions [69]. Mthatha is in a more rural setting compared to the other included metropoles and SWs there may have relatively less access to education and information. There is however, little data on SW sexual practices outside of large metropolitan areas.

MSM were most likely to have engaged in receptive anal sex in the past week, had relatively low reported use of condoms but high lubricant use and comparatively moderate substance use at last sex. High-risk sexual practices, particularly low levels of condom use have been identified among MSM in South Africa [70]. HIV prevention services for MSM have been operational in South Africa for almost a decade in the cities where MSM were recruited from, where condoms and lubricant are distributed [70]. Pre-exposure prophylaxis (PrEP) for MSM is available from the clinics where MSM were recruited, however, experience of PrEP was not assessed, which may have influenced condom use.

PWUD/ID were least likely to have engaged in sexual activity in the last month and reported relatively high condom use at last sex. Surprisingly, substance use at last sex was moderate and comparable to MSM, however, due to the frequency of substance use among PWUD/ID with a substance use disorder, under reporting may have taken place. While data on sexual practices among PWUD/ID in South Africa is limited, international research reflects that sexual practices are influenced by a range of factors, including relationship status [71] and mental health.

### Limitations

This paper is limited to a description of the findings, without in-depth analysis. Additional analysis around HBV, HCV and HIV infection and risks factors among PWID has been published elsewhere [28].

Several of the limitations and biases described here are inherent in research that is implemented in the context of programmatic service delivery in a way that minimizes impact on clients and staff.

This study drew on individuals already accessing available HIV and related prevention and treatment services and was also limited to a few of the sites and cities with existing targeted health services for KPs 6.

The selection bias associated with convenience sampling limits extrapolation to other members of the included populations. The findings may not be applicable to KPs with less access to health services, KPs who access services in the private healthcare sector, KPs with different risk profiles, or to KPs from other cities or regions in the country. Another important KP group that was not addressed by this study were prisoners in whom HBV, HCV and HIV prevalence in other global settings is known to be higher than in the general population [68, 72, 73].

Categorisation bias may have taken place in relation to same sex practices among male SWs and injecting drug use among MSM. However, no notable differences were identified among male SWs reporting recent anal sex and the prevalence of HIV and HBV (no SWs had HCV infection) compared to MSM. The higher HIV prevalence among the few MSM with a recent history of injecting is potentially linked to selection bias from MSM HIV and sexual health clinics, and the likelihood of them engaging in different sexual practices. The likely lower injection frequency among MSM who inject may have contributed to the lower prevalence of HBV and HCV identified among MSM with recent injection history compared to male PWUD/ID. Differences in type of drug injected and injecting frequency suggest that viewing these groups separately is useful.

The study did not comprehensively assess all the risk factors for blood borne infections, and did not include an assessment of knowledge or attitudes related to HIV and viral hepatitis.

The use of English for the questionnaire in a context of multiple different first languages (including Afrikaans, Xhosa and Zulu), and potential ad hoc translating by researchers, may have undermined accuracy of answers in the questionnaire.

Long-standing relationships between participants and research implementation organisations is likely to have impacted on reporting accuracy. However, reporting bias may have resulted in under- or mis-reporting of measures assessing substance use and sexual activity. This may have been especially marked in relation to activities generally associated with the KP the participant was not identifying as (for example, SWs may have been less comfortable reporting substance use than people identifying as PWUD/ID). Self-protective behaviours encouraged by implementing organizations (such as the use of sterile injecting equipment among PWUD/ID or condoms across all KPs) may also have been over-reported, resulting in an under-representation of risk practices, and an over-representation of harm reduction practices. The questionnaire did not assess frequency of use of the various substances enquired about thus limiting the insights into substance use within these populations.

Additional research is needed on the epidemiology of these infections among hard to reach PWUD/ID not routinely accessing mobile health services including those who may belong to other KPs or have additional risk factors, such as within the correctional service system. Further understanding of whether, and to what degree, knowledge of these infections and their associated risk factors among KP affects risk practices and prevalence would be valuable in the design of prevention and care services. Greater insights are also needed into factors associated with on-going parenteral risk in the presence of harm reduction services.

Pilot projects that explore innovative ways to provide integrated HBV-HCV-HIV and other holistic services to KPs in the South African context are needed to inform the granular detail and implementation experience that will be necessary for an effective integrated HBV/HCV/HIV response. Future research is needed to understand health care providers’ knowledge around viral hepatitis and HIV among at risk KPs to complement the existing epidemiological data.

## Conclusions

HBV, HCV and HIV pose major health threats for KPs in South Africa. Current HCV prevalence in PWUD/ID will increase in the absence of comprehensive HCV prevention services that include opioid substitution therapy and needle syringe services and a rapid, targeted and acceptable treatment programme. Similarly, the risk of HCV and HIV transmission needs to be addressed among MSM.

HIV prevalence remains high across all KPs. The fact that almost a fifth of HIV infected SWs are not on treatment indicates the need for tailored care provision based on an assessment of care access barriers as well as active education around transmission of blood borne viruses.

Eliminating viral hepatitis as a public health threat in South Africa by 2030 will only be an attainable reality once viral hepatitis prevention, testing and treatment services are provided to all high-risk KPs. Similar to dedicated MSM sexual health clinics, we need to provide appropriate healthcare services for SWs and PWUD/ID, ensuring access to sterile injecting equipment and opioid substitution therapy as needed. HBV, HCV and HIV screening, preferably with easily accessible and affordable POC diagnostics and seamless initiation of antiviral therapy should be standard of care. Like many other countries, South Africa has developed national guidelines for the management of viral hepatitis and even has a costed national action plan to address this issue, but political support, appropriate resource allocation and dedicated and passionate people, that include KPs, will be required to support implementation to achieve this ambitious goal.

## Data Availability

The datasets used and/or analysed during the current study are available from the corresponding author on reasonable request.

## Abbreviations

AIDS: Acquired Immunodeficiency Syndrome
ATS: Amphetamine-type stimulants
CPT: Cape Town
CI: Confidence Interval
CT: Cape Town
DAA: Direct Acting Antiviral
DBN: Durban
ELISA: Enzyme-linked Immunosorbent Assay
HBsAg: HBV Surface Antigen
HBV: Hepatitis B Virus
HCV: Hepatitis C Virus
HIV: Human Immunodeficiency Virus
IQR: Inter quartile range
JHB: Johannesburg
KP: Key Population
MSM: Men who have sex with men
NICD: National Institute for Communicable Diseases
OST: Opioid Substitution Therapy
PE: Port Elizabeth
POC: Point-of-care
PCR: Polymerase chain reaction
PMB: Pietermaritzburg
PTA: Pretoria
PWUD/ID: People who use drugs, including people who inject drugs
PWID: People who inject drugs
RNA: Ribonucleic acid
SW: Sex worker
NDOH: South African National Department of Health
UI: Uncertainty interval
WHO: World Health Organization

## Footnotes

1 Personal communication with Dr. Kgomotso Vilakazi Nhlapo, hepatitis lead at the South African National Department of Health on 29 April 2020.

2 At the time of study implementation TB HIV Care operated HIV prevention services for SWs (in Cape Town, Port Elizabeth, Durban, Pietermaritzburg) and PWUD/ID (in Cape Town and Durban) through mobile outreach and from community based health centres. OUT Wellbeing provided HIV prevention and treatment services to PWUD/ID and MSM in Pretoria through mobile outreach and from a community based health centre. Anova Health Institute provided HIV prevention and treatment services to MSM in Cape Town and Johannesburg from community based health centres.

3 The sites which recruited sex workers routinely provided services to people who accessed services tailored towards people who identify as sex workers, but no specific distinction was made between transactional sex and sex work.

4 This decision was made based on the acceptable safety profile and tolerance of HBV vaccination and insufficient funds for additional HBV testing.

5 Personal communication with Leora Casey, key population programme manager at NACOSA, 19 June 2019.

6 At the time of study implementation HIV prevention, sexual health and harm reduction services for KPs existed in several other locations that were not part of this study. Notably, SW services existed in major urban areas like Johannesburg, Ekurhuleni and Bloemfontein. MSM services existed in Durban, Pietermaritzburg, Bloemfontein, Kimberly and Ehlanzeni. PWUD/ID harm reduction services were started in Port Elizabeth and Johannesburg after the study started.
